# Impact of Inflammation and Muscle Mass on Prognosis in Hospitalized Patients with Suspected Dysphagia at a Tertiary Hospital

**DOI:** 10.3390/geriatrics11020042

**Published:** 2026-04-09

**Authors:** Mario Alfredo Saavedra-Vásquez, Juan José López-Gómez, Beatriz Ramos-Bachiller, Olatz Izaola-Jauregui, Eva López-Andrés, Isabel Pérez-Mellén, Sara Cuenca-Becerril, María Jesús Villameriel-Galván, Jaime González-Gutiérrez, Lucia Estevez-Asensio, María Ángeles Castro-Lozano, Daniel Antonio De Luis-Román

**Affiliations:** 1 Servicio de Endocrinología y Nutrición, Hospital Clínico Universitario de Valladolid, 47003 Valladolid, Spain; 2 Health Research Institute of Valladolid (IBioVALL), 47010 Valladolid, Spain; 3 Centro de Investigación de Endocrinología y Nutrición, Facultad Medicina, Universidad de Valladolid, 47005 Valladolid, Spain; 4 Centro de Investigación Biomedica en Red (CIBEROBN) de la Obesidad y Nutrición, Instituto de Salud Carlos III, 28029 Madrid, Spain

**Keywords:** hospitalized patients, mortality, hospital stay, muscle mass, inflammation

## Abstract

**Background/Objectives**: Dysphagia is associated with an increased risk of in-hospital complications and adverse outcomes. Prognosis in frail hospitalized populations is influenced by systemic inflammation and reduced muscle mass. Calf circumference (CC) and an estimated appendicular skeletal muscle index (ASMI) can serve as indirect measures of muscle mass, while inflammatory status may be captured by C-reactive protein (CRP), albumin, and the CRP/albumin ratio. This study aimed to evaluate the prognostic value of indirect biomarkers of inflammation and muscle mass to predict prognosis in hospitalized patients with suspected dysphagia. **Methods**: A retrospective observational study was conducted at a tertiary hospital and included patients admitted with suspected dysphagia between April 2015 and October 2024. On admission, demographic variables (sex and age), anthropometry (weight, height, and CC), EAT-10 (Eating Assessment Tool) score, and serum laboratory parameters (CRP, albumin) were collected. ASMI was estimated using the formula −10.427 + (CC × 0.768) − (age × 0.029) + (sex × 7.523)/(height^2^). Outcomes were in-hospital mortality and length of hospital stay. Comparisons were performed between survivors and non-survivors, and multivariable models adjusted for age and sex were used to identify independent associations with mortality. **Results**: A total of 4241 patients were included (51.2% women), with a median age of 85 (Interquartile range [IQR] 14) years and a mean EAT-10 score of 15.98 (SD 7.79). In-hospital mortality was 18.13% (*n* = 769). Non-survivors were older (86 [IQR 11] vs. 84 [IQR 14] years; *p* < 0.001) and displayed a more inflammatory profile, with higher CRP (78.1 [IQR 114.28] vs. 44 [IQR 96] mg/L) and CRP/albumin ratio (27.27 [IQR 43.04] vs. 13.64 [IQR 31.77]; *p* < 0.001), and lower albumin (3 [IQR 0.8] vs. 3.3 [IQR 0.8] g/dL; *p* < 0.001). They also had lower muscle mass, with reduced CC and lower ASMI in both sexes. In multivariable analysis, a higher CRP/albumin ratio was independently associated with increased odds of death (OR 1.011; 95% CI 1.008–1.014; *p* < 0.001), whereas a higher ASMI was protective (OR 0.885; 95% CI 0.801–0.978; *p* = 0.017). Higher CRP/albumin ratios were also associated with longer hospital stays and lower albumin, CC, and ASMI values. **Conclusions**: In hospitalized patients with suspected dysphagia, systemic inflammation and lower muscle mass were associated with worse clinical outcomes. The CRP/albumin ratio independently predicted higher in-hospital mortality and prolonged hospitalization, whereas higher estimated ASMI was associated with lower mortality risk, supporting the combined prognostic value of inflammatory and muscle-mass indicators in this population.

## 1. Introduction

Dysphagia is a swallowing disorder that compromises the safety and efficiency of swallowing and increases the risk of malnutrition, dehydration and other complications such as bronchoaspiration, which can aggravate the clinical condition, worsen the prognosis and reduce quality of life [1]. The prevalence of dysphagia increases with age; this age-related condition is known as presbyphagia and can affect up to 30% of the elderly population. This figure increases during hospitalization, reaching almost 50% due to the addition of other factors [2].

This represents a major clinical problem, especially due to its nutritional implications, as it can lead to lower intake due to the need to modify textures, avoid certain foods and limit volumes for safety reasons [1]. In turn, malnutrition and sarcopenia can aggravate dysphagia by compromising the strength and coordination of the oropharyngeal muscles, perpetuating a vicious cycle of low intake and functional deterioration. Furthermore, malnutrition on admission is one of the predictors of risk of complications that could result in increased hospitalization time, readmissions, poor response to treatment and higher in-hospital mortality [3].

In hospitalized patients, nutritional biomarkers may have limitations because acute inflammatory responses and changes in fluid balance alter both analytical parameters and anthropometric measurements [4]. Albumin, which is synthesized by the liver, acts not only as a nutritional marker but also as a negative acute-phase reactant [4]. Low albumin counts have been found to be associated with worse morbidity and mortality and prolonged hospital stays [5]. Systemic inflammation, involved in catabolic processes, also causes low albumin levels and functional impairment [4,6]. C-reactive protein (CRP) is a sensitive marker of inflammation, and its elevation is associated with an increased risk of adverse outcomes, including all-cause mortality [7]. However, the combination of these two parameters in the form of the CRP/albumin ratio has gained increasing relevance as a prognostic marker in hospitalized patients [8,9].

Furthermore, it has also been demonstrated that muscle mass acts as a physiological reserve and that its loss is associated with poorer clinical outcomes, making its preservation essential as it allows patients to better cope with the catabolism induced by hospitalization and immobility [10,11]. Anthropometric measurements serve as alternatives to imaging techniques for assessing muscle mass. Calf circumference (CC) is a simple, low-cost anthropometric measure that has been linked to length of hospital stay and mortality in hospital cohorts [12,13]. In addition, appendicular muscle mass estimated using predictive equations based on CC can be used to determine the Appendicular Skeletal Muscle Index (ASMI) in the absence of instrumental methods [14].

During hospitalization, it is particularly important to have simple markers that help assess both inflammatory burden and nutritional status, including muscle mass. Acute inflammation can contribute to the loss of this musculoskeletal reserve, especially in older hospitalized patients [15]. Similarly, even in the general population, higher CRP levels have been found to be associated with low muscle mass, reinforcing the idea that systemic inflammation and muscle deterioration often go hand in hand, beyond acute conditions [15,16]. For all these reasons, jointly evaluating inflammatory markers (CRP, albumin and CRP/albumin) and muscle mass measurements (CC and estimated ASMI) could provide additional and useful prognostic information in hospitalized patients with suspected dysphagia. To the best of our knowledge, no previous study has simultaneously evaluated the CRP/albumin ratio and estimated ASMI derived from calf circumference as joint prognostic markers in hospitalized patients with suspected dysphagia.

The aim of this study is to analyze the impact of systemic inflammation and muscle mass status on the prognosis of hospitalized patients with suspected dysphagia. We hypothesized that a higher CRP/albumin ratio and a lower estimated ASMI would be associated with worse in-hospital prognosis, reflected by increased mortality and prolonged hospital stay. Our goal was to identify simple and readily available clinical markers that allow for better risk stratification and early optimization of nutritional and functional interventions during hospitalization.

## 2. Materials and Methods

### 2.1. Study Design

This was a single-center retrospective cohort study of patients who were prescribed a specific dysphagia diet during their hospitalization at the University Clinical Hospital of Valladolid (Valladolid East Area) and assessed by the Clinical Nutrition Unit of the Endocrinology and Nutrition Service from 13 April 2015 to 15 October 2024. Prescription of a dysphagia diet by the attending medical team was considered an indicator of suspected dysphagia, and the patient was included in the study with an ‘EAT-10’ (Eating Assessment Tool) test greater than or equal to 3 points. Nutritional screening was performed during the first visit, as well as an initial nutritional assessment.

The study was conducted in accordance with current legislation and approved by the institutional ethics committee. When collecting data from medical records, the data were de-identified and anonymized, coding it in such a way that it was impossible to correlate the study results with the patient data. This study was approved by the Medical Research Ethics Committee (CEIm) of Valladolid Health Areas on 28 May 2025 with approval code PI-25-318-C.

### 2.2. Patient Selection

The patient selection criteria included adults aged 18 years or older who were admitted to the University Clinical Hospital of Valladolid and prescribed a dysphagia diet, with an EAT-10 score of 3 points or higher, between 13 April 2015 and 15 October 2024. In our institution, all patients for whom a dysphagia diet is prescribed are systematically referred to the Clinical Nutrition Unit according to a hospital protocol. Patients were excluded if they had not been assessed by the Clinical Nutrition Unit during their hospital stay. Verbal informed consent was obtained from the participants. Verbal consent was chosen instead of written consent due to the patients’ clinical condition during hospitalization and because the procedures were part of routine clinical practice. This approach was approved by the Ethics Committee, in accordance with applicable national legislation and institutional policies for retrospective chart-review studies.

### 2.3. Variables

#### 2.3.1. Clinical and Epidemiological

The cause of admission was categorized as follows: respiratory infection, non-respiratory infection, cardiological, neurological, traumatological, oncological, metabolic cause and other causes. Gender and age in years were also recorded.

In-hospital mortality and length of hospital stay were assessed. Short hospitalizations were defined as those lasting 6 days or less, and a prolonged stay as 16 days or longer. Because length of stay showed a non-normal distribution with extreme values, short and prolonged hospital stays were defined using the 25th and 75th percentiles of the sample distribution, respectively. This percentile-based approach was chosen to compare clinically distinct groups at both extremes of hospital stay and was considered more informative than a median split in this context.

#### 2.3.2. Anthropometry

Body weight was determined in kilograms (kg) using a calibrated scale, with the patient wearing light clothing and no footwear. Height was measured in meters (m) using a standardized height gauge, with the subject standing barefoot in an upright position, with heels together and head in the Frankfurt plane. Body mass index was calculated as weight in kilograms divided by height in meters squared (kg/m^2^).

For patients who could not be weighed and measured due to mobility limitations, estimates were made based on the brachial circumference—on the non-dominant arm, relaxed, taking the midpoint between the acromion and olecranon—and the cubital distance—measuring the distance between the olecranon and the styloid process of the ulna with the forearm flexed at 90° in centimeters (cm) using a flexible, non-stretchable tape measure.

In addition, calf circumference (CC) was measured in centimeters (cm) at the widest point of the calf, with the patient in a sitting or supine position, with the muscles relaxed. Estimated appendicular skeletal muscle mass index (ASMI) was calculated using the following formula

(1) $$\frac{-10.427+[0.768\times calf\;circumference(cm)]-[0.029\times age(years)]+[7.523\times sex]}{\left[height(m)\times height(m)\right]}$$

where sex was coded as male = 1 and female = 0 [14].

#### 2.3.3. Biochemical Variables

Biochemical variables were analyzed using a Cobas c711 Autoanalyzer (Roche Diagnostics, Basel, Switzerland), following standardized laboratory procedures and internal quality control protocols. The nutritional and inflammatory parameters evaluated included serum albumin, measured in grams per deciliter (g/dL), as a marker of nutritional status and negative acute-phase reactant; C-reactive protein (CRP), measured in milligrams per liter (mg/L), as a sensitive indicator of systemic inflammation; and prealbumin, also expressed in mg/dL, which reflects short-term changes in protein status.

In addition, the CRP/albumin ratio was calculated as an integrated biomarker that provides complementary information on the interaction between inflammatory burden and nutritional impairment, allowing for a more comprehensive assessment of patients’ metabolic and inflammatory status at the time of hospital admission.

#### 2.3.4. EAT-10

The Eating Assessment Tool-10 (EAT-10) was administered, and the total score was recorded. This standardized instrument for the initial assessment of dysphagia consists of a 10-item questionnaire, where each item is scored from 0 (no problem) to 4 (severe problem), with a total range of 0 to 40 points. A score equal to or greater than 3 is considered abnormal and suggests a risk of dysphagia. This tool has demonstrated validity and reliability in multiple international studies, correlating with objective findings in tests such as videofluoroscopy and swallowing endoscopy [17,18,19].

### 2.4. Statistical Analysis

Statistical analysis was performed using IBM SPSS Statistics 23^®^ (SPSS Inc., Chicago, IL, USA), available at the Centre for Research in Endocrinology and Clinical Nutrition (CIENC)—University of Valladolid. Normality of continuous variables was assessed using the Kolmogorov–Smirnov test. Normally distributed variables are presented as mean ± standard deviation (SD) and were compared using the unpaired Student’s *t*-test, whereas non-normally distributed variables were presented as median (interquartile range [IQR]) and were compared using the Mann–Whitney U test to compare survivors and non-survivors, as well as patients with short and prolonged hospital stays. To classify hospital stays, cut-off points based on percentiles were used: short stay if the duration was less than the 25th percentile (p25) and long stay if it was greater than the 75th percentile (p75). Patients who died during hospitalization were excluded from the analyses of length of stay; therefore, short versus prolonged hospital stay was assessed only among survivors. Missing data were not imputed. Each analysis was performed using only cases with available data for the variables included in that specific analysis.

Given the limitations of CC, patients admitted for cardiac conditions and patients with a BMI ≥ 30 kg/m^2^ were excluded from the analysis of PC and calculated ASMI. Pearson’s or Spearman’s correlation coefficients were used as appropriate, according to variable distribution. Finally, a multivariate analysis was performed using binary logistic regression to identify factors independently associated with in-hospital mortality. Statistical significance was assessed using the *p*-value, with *p* < 0.05 considered relevant, and odds ratios (OR) with 95% confidence intervals (95% CI) were calculated to estimate the magnitude of the association.

## 3. Results

### 3.1. Sample Description

A total of 4241 patients were included, of whom 51.2% were women. All completed EAT-10 questionnaires had scores of 3 points or higher, with an average of 15.98 points (SD 7.79). The results broken down by sex are summarized in Table 1. For analyses involving CC and estimated ASMI, patients with BMI ≥ 30 kg/m^2^ and those admitted for cardiac causes were excluded. In the overall sample, there were 2068 men and 2173 women. After these exclusions, the subsample available for CC and estimated ASMI analyses comprised 1776 men and 1746 women.

Differences between sexes are shown in Table 1, with statistically significant differences between the two sexes in age, hospital stay, CRP, albumin, CRP/albumin ratio, and CC.

**Figure 1:**
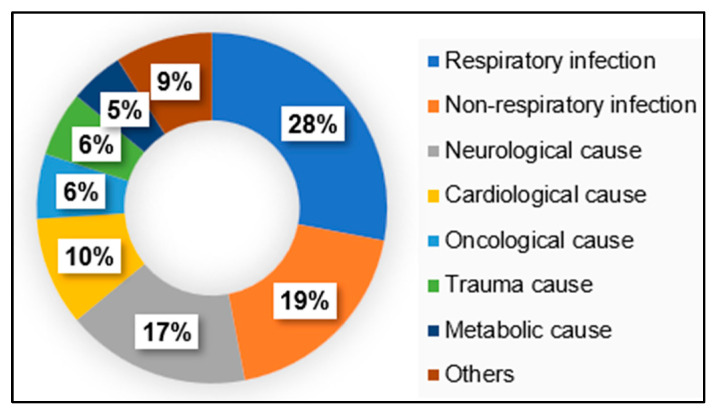
Causes of hospital admission.

Figure 1 shows the distribution of patients according to the cause of admission, with infectious conditions—particularly respiratory infections—being the most prevalent, followed by neurological and cardiological causes, among others.

### 3.2. Differences in Mortality Rates

Patients who died during hospitalization represented 18.13% of the study population. These patients were older and had significantly higher CRP and CRP/albumin ratio values, as well as lower albumin, CC and ASMI values (Table 2).

In the multivariate analysis using binary logistic regression, the highest CRP/albumin ratio (OR 1.011; 95% CI: 1.008–1.014; *p* < 0.001) was a risk factor and the highest ASMI (OR 0.885; 95% CI: 0.801–0.978, *p* = 0.017) was a protective factor independently associated with death, adjusted for sex and age.

### 3.3. Differences According to Length of Stay

A short stay was defined as <6 days (p25) and a prolonged stay was defined as >16 days (p75). Patients with prolonged hospital stay had significantly higher values for BMI, CRP, CRP/albumin ratio, and CC/ASMI (only in females). On the other hand, this group had statistically significantly lower values for age and albumin (Table 3).

### 3.4. Correlation Analysis

A positive correlation was observed between the length of hospital stay and CRP values (ρ = 0.07; *p* < 0.001) and the CRP/albumin ratio (ρ = 0.083; *p* < 0.001), as well as a negative correlation between length of stay and albumin values (ρ = −0.139; *p* < 0.001) in a statistically significant manner. No statistically significant correlation was observed between length of stay and CC in either males (r = −0.003; *p* = 0.926) or females (r = −0.13; *p* = 0.638), nor with ASMI in males (r = −0.028; *p* = 0.334) or women (r =−0.04; *p* = 0.879).

Finally, significant negative correlations were observed between the CRP/Albumin ratio and CC and ASMI in both men (r = −0.229; *p* < 0.001 and r = −0.222; *p* < 0.001, respectively) and women (r = −0.122; *p* < 0.001 and r = −0.129; *p* < 0.001, respectively), reaching statistical significance.

## 4. Discussion

This was a single-center retrospective cohort study, conducted in patients admitted with suspected dysphagia, which evaluated the impact of inflammation and muscle mass on patient prognosis using simple and accessible clinical markers. We found that patients who died during hospitalization were older, showed an inflammatory profile (higher CRP and CRP/albumin ratio values, as well as lower serum albumin levels), and had lower muscle mass, reflected by lower CC and ASMI values. Mean calf circumference values in both men and women were below the diagnostic thresholds for malnutrition, although the cut-off points differed by sex. Patients with prolonged hospital stays also showed greater inflammation, reflected by higher CRP and CRP/albumin ratio values and lower albumin levels. Higher CC and ASMI values were observed only in women. The inverse relationship between inflammation and muscle mass was supported by the negative correlations observed between these variables. Finally, multivariate analysis confirmed the risk associated with a high CRP/albumin ratio and the protective effect of a higher ASMI value against in-hospital mortality, after adjusting for sex and age.

The vast majority of patients in our cohort (87.4%) had a low ASMI compared to the cut-off points of 5.5 kg/m^2^ for women and 7.0 kg/m^2^ for men [20,21]. This proportion was higher than in the study by Miyahara S et al., which studied ASMI estimated using different formulas in hospitalized patients over 65 years of age (between 66.2% and 85.7% depending on the estimation method), although patients with obesity or admitted for cardiac reasons, in whom there could be an increase in CC/ASMI estimated due to adiposity and oedema, were not excluded [22].

Patients who died during hospitalization had significantly lower CC and estimated ASMI values than survivors, confirming through multivariate analysis adjusted for sex and age that a higher ASMI value acted as a protective factor against mortality. A previous systematic review and meta-analysis demonstrated that reduced ASMI is significantly associated with increased mortality in older adults, consistent with our findings [22,23]. Similarly, a Brazilian study by Maffini LF et al. found an increased risk of mortality due to low CC according to a cut-off point of ≤33 cm for women and ≤34 cm for men (OR = 4.1; 95% CI 1.3–13.0) [13].

Our study found no correlation between muscle mass markers (CC and ASMI) and hospital stay. In addition, significantly higher CC and ASMI were observed in women with prolonged hospital stays. This may be explained by the fact that prolonged hospital stays are often driven more by associated comorbidities, acute in-hospital complications, or social and care-related factors than by muscle mass alone. Consequently, the lack of correlation could indicate that these markers do not fully reflect the clinical complexity of hospitalized patients [22].

By contrast, inflammation was assessed using laboratory parameters such as elevated CRP values and the CRP/albumin ratio, and low albumin values, which were significantly associated with both prolonged hospital stays and mortality. A previous study showed that both high CRP levels (≥5 mg/dL) and low albumin levels (<3.5 g/dL) were associated with the risk of in-hospital death, which is in line with our findings [24]. We confirmed that the CRP/albumin ratio, using multivariate analysis adjusted for sex and age, acted as a risk factor for mortality, as seen in previous studies [7,8,9].

These markers are unlikely to be useful as standalone prognostic tools, but they may complement existing geriatric and nutritional risk-stratification approaches. Integrating them with existing geriatric and nutritional assessment frameworks by combining information on inflammatory burden and muscle reserve, particularly in hospitalized patients with suspected dysphagia, in whom rapid and accessible risk stratification is needed.

A major strength of this study is the large sample size, comprising more than 4000 patients, which substantially enhances the statistical power and external validity of the findings. In addition, the formula used to estimate ASMI relies solely on variables routinely recorded in medical charts—namely, anthropometric data, sex, and age—without the need for additional procedures or specialized measurement techniques, thereby supporting its feasibility and applicability in everyday clinical practice.

Although methods such as dual-energy X-ray absorptiometry (DXA) are recommended for assessing muscle mass, their use in routine clinical practice is often limited due to availability, cost and logistics, highlighting the need for simpler and more accessible alternative tools. In this context, despite the proven validity of calf circumference as an indirect marker of muscle mass, a previous study showed that muscle mass was assessed in only 13% of patients, highlighting a significant underestimation in healthcare practice [25].

This study has several limitations. First, it was conducted in a population with a high suspicion of dysphagia; therefore, caution is warranted when extrapolating the findings to other clinical settings or populations. Nevertheless, given that muscle mass assessment is particularly relevant in this group because of the elevated risk of sarcopenia, the validation of muscle mass estimation equations in this context represents a meaningful contribution. In this regard, it should also be acknowledged that calf circumference (CC) has inherent limitations as a surrogate marker of muscle mass, as adiposity and oedema may lead to an overestimation in individuals with obesity or fluid retention, thereby complicating clinical interpretation. To mitigate this potential bias, patients admitted for cardiac conditions and those with a body mass index ≥ 30 kg/m^2^ were excluded from the analysis. Because of the retrospective design, DXA or ultrasound measurements were not available in our cohort, precluding internal validation of estimated ASMI. Nevertheless, the equation used to estimate muscle mass was derived from a previous study developed with DXA as the reference method, in which the best-performing model showed high agreement with DXA-measured ASMI [14].

Second, as the primary aim of the study was to evaluate the prognostic impact of estimated muscle mass and inflammatory status at hospital admission, it was not feasible to adjust for all potential confounding factors. Variables such as nutritional interventions, changes in pharmacological treatment, mobilization during hospitalization, additional comorbidities, acute in-hospital complications, and social or care-related factors may also influence prognostic outcomes and could not be fully accounted for in the present analysis. For all these reasons, the study results should be interpreted as a prognostic model adjusted for a limited set of a priori selected variables, rather than as a fully adjusted causal model.

Third, because of the retrospective database design, repeated admissions of the same patient may have occurred and were analyzed as independent hospitalization episodes. In addition, some variables had missing data. No imputation, sensitivity analyses, or formal assessment of the missingness pattern were performed; therefore, potential bias related to non-random missingness cannot be excluded.

## 5. Conclusions

This observational study demonstrated that a higher ASMI—estimated using a formula based on the patient’s age, sex, and anthropometry—was significantly associated with lower in-hospital mortality. Given the simplicity of its calculation and the fact that it does not require specialized equipment, this equation may be a practical and easily applicable alternative for assessing muscle mass in a wide variety of hospitalized patients with a high suspicion of dysphagia.

Furthermore, inflammation, as assessed by routine laboratory markers, was significantly associated with mortality. When considering the effects of systemic inflammation and malnutrition together, the CRP/albumin ratio was identified as an independent risk factor for mortality. Because this index is derived from laboratory tests routinely available at hospital admission, it may represent a cost-effective and accessible tool for early risk stratification in this patient population.

Overall, early identification of these short-term risk markers in hospitalized patients can facilitate the implementation of closer monitoring, individualized care planning, and the timely adoption of nutritional and/or anti-inflammatory interventions during the initial and critical phase of hospitalization.

Future studies should examine the inflammatory status measured in our study using analytical markers and the muscle mass inferred using anthropometric measurements with techniques such as artificial intelligence-assisted muscle ultrasound, which allows both muscle quantity and quality to be assessed in real time and non-invasively. Another relevant line of research is to determine how acute and chronic inflammation affects functional recovery and the progression of dysphagia, as well as the development of personalized nutritional and pharmacological interventions based on the patient’s inflammatory profile.

## Figures and Tables

**Table 1:** Baseline characteristics by sex.

	Global	Female	Male	*p*
Frequency	4241	2173	2068	
Age (years)	85 (IQR 14)	87 (IQR 10)	82 (IQR 14)	<0.001
Hospital stay (days)	10 (IQR 10)	9 (IQR 9)	10 (IQR 10)	<0.001
BMI (kg/m^2^)	23 (IQR 5.47)	22.9 (IQR 5.83)	23 (IQR 4.91)	0.118
CRP (mg/L)	49 (IQR 102.88)	42 (IQR 95)	57 (IQR 108.8)	<0.001
Albumin (g/dL)	3.26 (IQR 0.7)	3.3 (IQR 0.7)	3.2 (IQR 0.8)	0.011
CRP/Albumin	15.68 (IQR 35.02)	12.92 (IQR 31.92)	18.24 (IQR 38.83)	<0.001
CC (cm)	-	28.11 (SD 3.67)	29.22 (SD 3.65)	<0.001
ASMI (kg/m^2^)	-	3.6 (SD 1.18)	6.13 (SD 1.07)	-
Normal ASMI according to sex	12.6%	5.4% (>5.5)	19.8% (>7)	12.6%

BMI: Body Mass Index, CRP: C-reactive protein, CC: calf circumference, ASMI: Appendicular Skeletal Muscle Mass Index, IQR: interquartile range, SD: standard deviation. Analyses involving CC and estimated ASMI were performed in the subsample excluding patients with BMI ≥ 30 kg/m^2^ and cardiac admissions.

**Table 2:** Comparison between deceased and non-deceased patients.

	Deceased	Non-Deceased/Alive	*p*
*n* (%)	769 (18.13%)	3472 (81.87%)	
Age (years)	86 (IQR 11)	84 (IQR 14)	<0.001
CRP (mg/L)	78.1 (IQR 114.28)	44 (IQR 96)	<0.001
Albumin (g/dL)	3 (IQR 0.8)	3.3 (IQR 0.8)	<0.001
CRP/Albumin	27.27 (IQR 43.04)	13.64 (IQR 31.77)	<0.001
Female CC (cm)	27.56 (SD 3.61)	28.2 (SD 3.67)	0.019
Female ASMI (kg/m^2^)	3.4 (SD 1.18)	3.63 (SD 1.18)	0.012
Male CC (cm)	28.46 (SD 3.36)	29.37 (SD 3.68)	<0.001
Male ASMI (kg/m^2^)	5.9 (SD 0.99)	6.17 (SD 1.08)	<0.001

CRP: C-reactive protein, CC: calf circumference, ASMI: Appendicular Skeletal Muscle Mass Index, IQR: interquartile range, SD: standard deviation. Analyses involving CC and estimated ASMI were performed in the subsample excluding patients with BMI ≥ 30 kg/m^2^ and cardiac admissions.

**Table 3:** Comparison between short and prolonged hospital stays.

	<6 Days	>16 Days	*p*
Age (years)	86 (IQR 12.25)	80 (IQR 17)	<0.001
CRP (mg/L)	29.45 (IQR 77.92)	46 (IQR 100.65)	<0.001
Albumin (g/dL)	3.4 (IQR 0.7)	3.2 (IQR 0.8)	<0.001
CRP/Albumin	8.7 (IQR 24.99)	14.84 (IQR 35.82)	<0.001
Female CC (cm)	27.74 (SD 3.72)	29.15 (SD 3.91)	<0.001
Female ASMI (kg/m^2^)	3.48 (SD 1.19)	3.92 (SD 1.23)	<0.001
Male CC (cm)	29.54 (SD 3.96)	29.86 (SD 3.33)	0.493
Male ASMI (kg/m^2^)	6.23 (SD 1.11)	6.27 (SD 1.06)	0.733

CRP: C-reactive protein, CC: calf circumference, ASMI: Appendicular Skeletal Muscle Mass Index, IQR: interquartile range, SD: standard deviation. Analyses involving CC and estimated ASMI were performed in the subsample excluding patients with BMI ≥ 30 kg/m^2^ and cardiac admissions. Short versus prolonged hospital stay was assessed only among survivors.

## Data Availability

Data is unavailable due to privacy and ethical restrictions; however, it can be provided upon request from the authors.
